# Discrimination and learning of temporal input sequences in a cerebellar Purkinje cell model

**DOI:** 10.3389/fncel.2023.1075005

**Published:** 2023-02-02

**Authors:** Kaaya Tamura, Yuki Yamamoto, Taira Kobayashi, Rin Kuriyama, Tadashi Yamazaki

**Affiliations:** ^1^Graduate School of Informatics and Engineering, The University of Electro-Communications, Tokyo, Japan; ^2^Graduate School of Medical and Dental Sciences, Tokyo Medical and Dental University, Tokyo, Japan; ^3^Graduate School of Sciences and Technology for Innovation, Yamaguchi University, Yamaguchi, Japan

**Keywords:** cerebellum, Purkinje cell, dendritic computation, direction sensitivity, spatiotemporal learning, discrimination

## Abstract

**Introduction:**

Temporal information processing is essential for sequential contraction of various muscles with the appropriate timing and amplitude for fast and smooth motor control. These functions depend on dynamics of neural circuits, which consist of simple neurons that accumulate incoming spikes and emit other spikes. However, recent studies indicate that individual neurons can perform complex information processing through the nonlinear dynamics of dendrites with complex shapes and ion channels. Although we have extensive evidence that cerebellar circuits play a vital role in motor control, studies investigating the computational ability of single Purkinje cells are few.

**Methods:**

We found, through computer simulations, that a Purkinje cell can discriminate a series of pulses in two directions (from dendrite tip to soma, and from soma to dendrite), as cortical pyramidal cells do. Such direction sensitivity was observed in whatever compartment types of dendrites (spiny, smooth, and main), although they have dierent sets of ion channels.

**Results:**

We found that the shortest and longest discriminable sequences lasted for 60 ms (6 pulses with 10 ms interval) and 4,000 ms (20 pulses with 200 ms interval), respectively. and that the ratio of discriminable sequences within the region of the interesting parameter space was, on average, 3.3% (spiny), 3.2% (smooth), and 1.0% (main). For the direction sensitivity, a T-type Ca^2+^ channel was necessary, in contrast with cortical pyramidal cells that have *N-methyl-D*-aspartate receptors (NMDARs). Furthermore, we tested whether the stimulus direction can be reversed by learning, specifically by simulated long-term depression, and obtained positive results.

**Discussion:**

Our results show that individual Purkinje cells can perform more complex information processing than is conventionally assumed for a single neuron, and suggest that Purkinje cells act as sequence discriminators, a useful role in motor control and learning.

## 1. Introduction

Temporal information processing is ubiquitous in our daily lives, and ranges from sub-milliseconds for sound localization to days and weeks for memory consolidation (Mauk and Buonomano, 2004; Meck, 2005). In particular, the range of hundreds of milliseconds up to a few seconds is particularly important for motor control, where individual muscle contractions are orchestrated to achieve smooth and coordinated movement (Ito, 2012). For such motor control and motor learning, the cerebellum plays essential roles, and the involvement of the cerebellum in temporal information processing has been studied intensively using Pavlovian delay eyeblink conditioning (Ivry and Spencer, 2004). On the neural mechanisms of representation of the passage of time necessary for this task, various hypotheses have been proposed (e.g., Yamazaki and Tanaka, 2009 for review, also see Johansson et al., 2014). Most hypotheses propose that representation of the passage of time is realized by the network of neurons, for example, by the dynamics of the recurrent network composed of granule cells and Golgi cells in the granular layer (Buonomano and Mauk, 1994; Medina et al., 2000; Hofstötter et al., 2002; Yamazaki and Tanaka, 2005), rather than by individual neurons. Most studies have assumed that individual neurons are simple elements that cannot perform complex tasks.

On the contrary, recent experimental and computational studies have revealed powerful computational capabilities of single neurons by harnessing non-linearity of dendritic information processing and dynamics of various ion channels (Koch and Segev, 2003; London and Häusser, 2005). Such “dendritic computation” participates in even temporal information processing. In a pioneering computational study by Rall (1964), brief and sequential activation of small segments called compartments constituting a dendritic cable evoked different somatic responses depending on the direction of stimulation [e.g., from proximal to distal (IN) or distal to proximal (OUT)], suggesting that the dendritic cable is capable of direction sensitivity. In that study, the temporal interval for each pair of stimuli was up to a few milliseconds, and the duration for the entire stimulation was up to tens of milliseconds. This was due to the lack of active ion channels on dendrites (i.e., passive cables).

More recently, Branco et al. (2010) demonstrated both experimentally and computationally that similar sequential activation on a single dendrite with the duration of hundreds of milliseconds evoked different activity patterns in cortial layer 2/3 pyramidal neurons, where active ion channels, specifically *N*-methyl-*D*-aspartate receptors (NMDARs), and intracellular calcium played an essential role in the direction sensitivity. These results suggest that even individual neurons can exhibit capability of temporal information processing in the range of hundreds of milliseconds that are necessary for motor control.

In this study, we focused on dendritic computation by Purkinje cells (PCs) in the cerebellum, because the cells provide the sole outputs from the cerebellar cortex to the downstream deep cerebellar nuclei, and the cells have remarkably large dendrites expressing various ion channels. We examined if PCs can exhibit similar discrimination ability of input sequences that span in the temporal range appropriate for motor control. Specifically, we fed temporal sequences of short pulses as excitatory inputs from parallel fibers (PFs) to the PC's dendrites, and measured whether spikes were emitted at the soma in response to the excitatory inputs. Eventually, we conducted computer simulation of a biophysical cerebellar PC model, and observed similar direction sensitivity on sequential activation of multiple dendritic locations by brief pulses mimicking PF stimuli. Moreover, we investigated whether a cell can learn particular sequence of stimuli by spike-timing-dependent plasticity (STDP), which pairs a sequential stimulus and the injection of an instruction signal; this would support the idea that cerebellar PCs modify synaptic weights of PFs by the presence or absence of a climbing fiber (CF) input stimulus (Ito et al., 2014).

## 2. Materials and methods

### 2.1. Purkinje cell model

**Figure 1:**
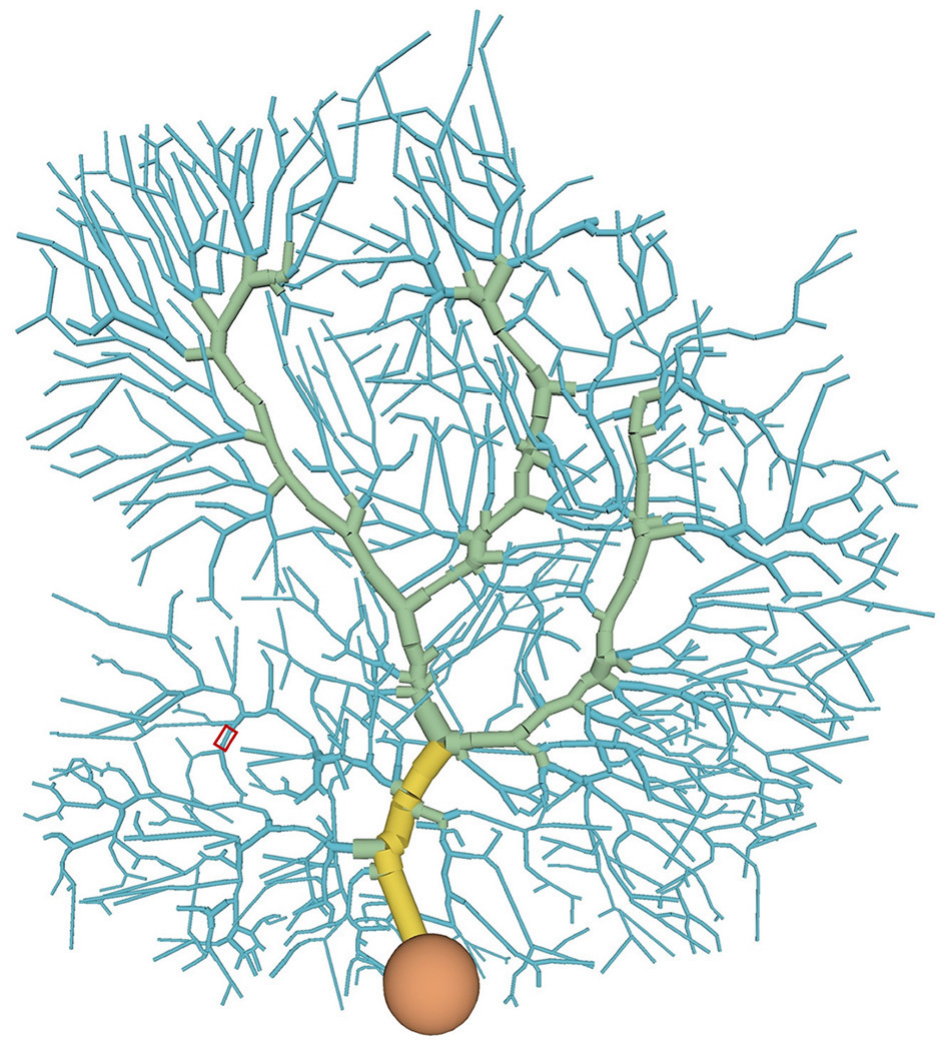
Distribution of compartment types in the PC model: soma (orange), main dendrite (yellow), spiny dendrite (blue), and smooth dendrite (green). Each type has different ion channels. A typical stimulated site was shown in a red box (see also Figure 4A).

We used a multi-compartment model of cerebellar PCs (De Schutter and Bower, 1994). The model consists of 1,600 compartments, which are classified into one of four types (soma, main dendrite, spiny dendrite, smooth dendrite) (Figure 1, Supplementary Video 1). Each compartment has several ion channels (Table 1).

**Table 1:** Ion channels of the PC model (De Schutter and Bower, 1994).

**Compartments**	**Number of compartments**	**Ion channels**
Soma	1	Leak, NaF, NaP, CaT, Kh1, Kh2, Kdr, KM, KA
Main dendrite	9	Leak, CaP, CaT, Kdr, KM, KA, KC, K2
Smooth dendrite	105	Leak, CaP, CaT, KM, KC, K2
Spiny dendrite	1,485	Leak, CaP, CaT, KM, KC, K2

Leak, Leak channel; NaF, Fast sodium channel; NaP, Persistent sodium channel; CaP, P-type calcium channel; CaT, T-type calcium channel; Kh, Anomalous rectifier channel; Kdr, Delayed rectifier channel; KM, Persistent potassium channel; KA, A-type potassium channel; KC, BK-type calcium-activated potassium channel; K2, K2-type calcium-activated potassium channel.

In this model, a membrane potential is described by a cable equation. For example, in the case of one dimensional cable, the equation is as follows:

(1) $$\begin{array}{l} c_{\text{m}}\frac{\partial v\left(t,x\right)}{\partial t}=-\frac{a}{2R}\frac{\partial^{2}v\left(t,x\right)}{\partial x^{2}}-\underset{\text{k}}{\sum }I_{\text{k},j}\left(v\left(t,x\right)\right)+I_{\text{ext}}\left(t,x\right), \end{array}$$

where *v*(*t, x*) is membrane potential at time *t* and position *x*, *c*_m_ is membrane capacitance, *a* is radius of a cable, *R* is intracellular resistance, *I*_k_ is ionic current of channel k, and *I*_ext_ is external current. Equation (1) is a type of partial differential equation. Because a computer cannot solve partial differential equations so Equation (1) is discretized about the first term of the right-hand side by taking a secondary central difference with respect to space *x* as follows:

(2) $$\begin{array}{l} c_{\text{m}}\frac{\partial v_{j}\left(t\right)}{\partial t}=-\frac{a}{2R}\frac{v_{j+1}\left(t\right)-2v_{j}\left(t\right)+v_{j-1}\left(t\right)}{\Delta x^{2}}-\underset{\text{k}}{\sum }I_{\text{k}}\left(v_{j}\left(t\right)\right)+I_{\text{ext},j}\left(t\right), \end{array}$$

where Δ*x* is the space width and *j* is the index of dis-cretized compartments. Equation (2) corresponds to a compartment. Therefore, the partial differential equation (Equation (1)) turns into a system of ordinary differential equations, called the methods of lines. Thus, a computer can solve these equations numerically.

Ionic currents are described by Hodgkin-Huxley type equations:

(3) $$\begin{array}{l} I_{\text{k},j}=g_{\text{k}}m^{q}h^{r}\left(v_{j}\left(t\right)-E_{\text{k}}\right), \end{array}$$

where *I*_k_ is the ionic current of channel k, *g*_k_ is a conductance of the ionic current of channel k, *m* is an activation variable, *h* is an inactivation variable, *E*_*k*_ is a reversal potential, and *q, r* are constants. Activation and inactivation variables *m* and *h*, which are called gate variables, develop temporally by the following equation:

(4) $$\begin{array}{l} \tau_{x}\frac{\partial x}{\partial t}=x_{\infty }\left(v\right)-x, \end{array}$$

where *x* is either *m* or *h*, τ_*x*_ is the time constant, and *x*_∞_ is the maximal value.

In five channels (fast sodium channel: NaF, persistent sodium channel: NaP, P-type calcium channel: CaP, T-type calcium channel: CaT, and A-type potassium channel: KA), *x*_∞_ and τ_*x*_ depend on α(*v*), β(*v*) as follows:

(5) $$\begin{array}{l} \begin{array}{c} x_{\infty }=\frac{\alpha \left(v\right)}{\alpha \left(v\right)+\beta \left(v\right)}\\ \tau_{x}=\frac{1}{\alpha \left(v\right)+\beta \left(v\right)}, \end{array} \end{array}$$

where α(*v*) and β(*v*) are defined for each channel (Supplementary Table S1). On the other hand, in the other channels, *x*_∞_ and τ_*x*_ are defined directly (Supplementary Table S2).

The PC model has its direct successor (Zang et al., 2020; Zang and De Schutter, 2021). The new model has more biological details and therefore exhibit more realistic behaviors. In particular, the model emits spikes spontaneously even without excitatory inputs. This behavior, however, makes examining the discrimination ability based on somatic spike responses difficult. Thus, we used the original model (De Schutter and Bower, 1994) in this study. We discussed this issue in the Limitations of the Discussion.

### 2.2. Stimulation of dendrites in the PC model

**Figure 2:**
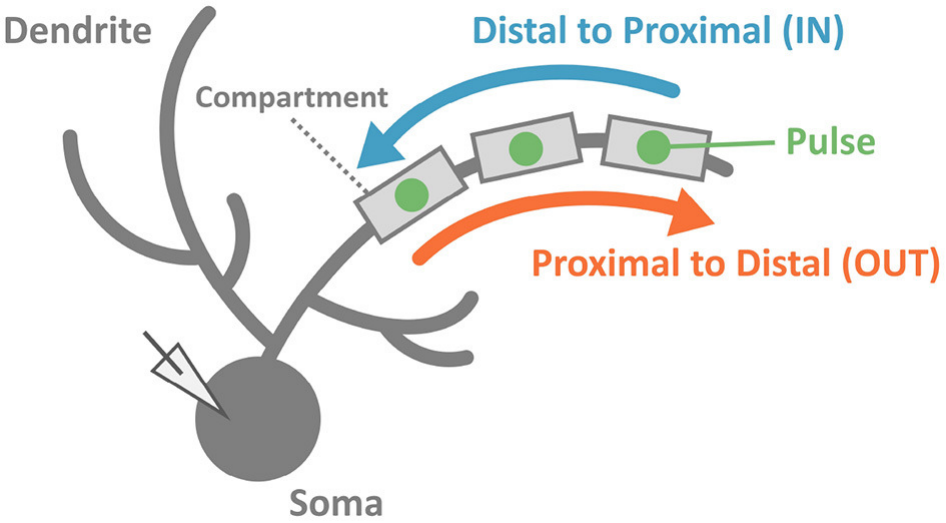
Schematic of stimulation and recording in the simulation. We injected two sequences consisting of multiple pulses with the same strength but different input orders (IN or OUT). Soma membrane potentials were recorded to investigate whether the PC model could discriminate spatiotemporally different pulse sequences. In this study, we considered spiking or not spiking in the soma for classifications of results.

To reveal the capability of discrimination for temporal input sequences (or just “sequences" for short) in PCs, we stimulated dendrites in various ways. We injected multiple short current pulses at various dendritic compartments at various timings. In a typical setting, we chose 5 compartments aligned in straight, and fed short pulses to them sequentially either from distal to proximal dendrites (IN direction) or from proximal to distal dendrites (OUT direction). Then, we examined whether the soma emit spikes (Figure 2).

A current pulse fed to compartment *j* was originally modeled as

(6) $$\begin{array}{l} I_{\text{ext},j}\left(t\right)=\{\begin{array}{cc} w_{j} & \left(0\leq t-t_{j}<1\text{ ms}\right)\\ 0 & \text{otherwise} \end{array}, \end{array}$$

where *I*_ext, *j*_(*t*) is the current injected to the *j*-th dendritic compartment, *t*_*j*_ is the time [ms] for the injection, and *w*_*j*_ is the pulse amplitude. The duration of each pulse was set at 1 ms. We assumed that pulses represent brief synaptic inputs at PFs, and so *w*_*j*_ represents the synaptic weight. However, due to the rapidly changing currents, numerical calculation became unstable by using Equation (6). To avoid numerical instability, we slowly ramped up the current amplitude as in Equation (7) instead.

(7) $$\begin{array}{l} I_{\text{ext},j}\left(t\right)=\{\begin{array}{cc} w_{j}\left(1-\exp\left(-\frac{t-t_{j}}{\tau_{I}}\right)\right) & \left(0\leq t-t_{j}<1\text{ ms}\right)\\ 0 & \text{otherwise} \end{array}, \end{array}$$

where τ_*I*_ is a time constant determining the ramp-up speed and was set at 50 ms due to numerical instability in the simulation.

We also varied the number of pulses (i.e., the number of stimulated compartments) from 2 to 20, synaptic weights from 1 to 10 nA while changing every 0.5 nA, and interval from 10 to 200 ms while changing every 10 ms.

### 2.3. Role of Ca^2+^ channels

**Figure 3:**
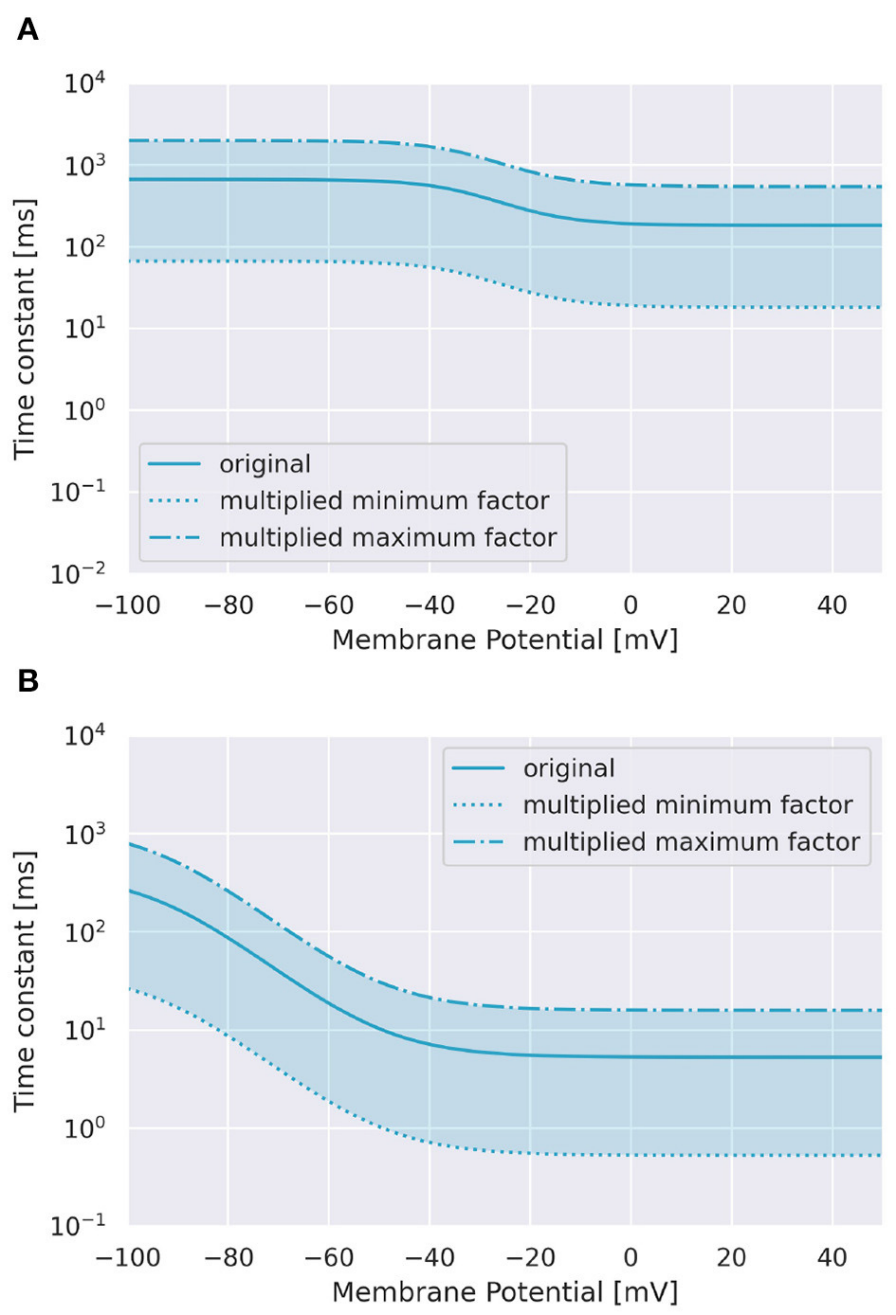
Range of inactivation time constants changed in the simulation. **(A)** CaP channel. **(B)** CaT channel.

To examine the role of Ca^2+^ channels on the discrimination, we changed the time constants of inactivation variables *h* of two Ca^2+^ channels in terms of memory; the P-type Ca^2+^ ion channel (CaP) and the T-type Ca^2+^ ion channel (CaT). Specifically, τ was multiplied by a factor *f* taking values from 0.1 to 3.0 (Figure 3). The number of pulses was 6, and synaptic weight was 6.633 nA.

### 2.4. Learning of sequence direction

To test whether the PC could learn to respond to a sequence with the reverse order, we mimicked long-term depression (LTD) at PF induced by the CF inputs. A stimulus with a certain direction and set of parameters was fed, and after 2 ms of the end of stimulation, we fed a paired simulated CF stimulus. Then, the synaptic weight of the PF was modified based of the time difference between the onset of the PF stimulation and that of the CF stimulation (i.e., STDP). We then examined whether the same stimulus or the stimulus in the reverse order was able to activate the PC.

For implementation of STDP, we defined trace *x*_*j*_ of PF at compartment *j*,

(8) $$\begin{array}{l} x_{j}\left(t\right)=\exp\left(-\frac{\Delta t}{\tau_{\text{pre}}}\right)x_{j}\left(t-\Delta t\right)+S_{j}\left(t\right), \end{array}$$

where *t* is time, τ_pre_ is a time constant, and *S*_*j*_ is either 0 or 1 representing a pulse input at compartment *j*. Similarly, trace *y* of CF was defined as:

(9) $$\begin{array}{l} y\left(t\right)=\exp\left(-\frac{\Delta t}{\tau_{\text{post}}}\right)y\left(t-\Delta t\right)+S\left(t\right), \end{array}$$

where τ_post_ is a time constant, and *S* is either 0 or 1 representing non-spiking and spiking, respectively. From Equation (8),(9), the STDP rule is described as follows:

(10) $$\begin{array}{l} \begin{array}{l} w_{j}\left(t+\Delta t\right)=w_{j}\left(t\right)+\Delta w_{j}\left(t\right)\\ \;\Delta w_{j}\left(t\right)=-A_{1}y\left(t\right)S_{j}\left(t\right)+A_{2}x_{j}\left(t\right)S\left(t\right)+A_{3}, \end{array} \end{array}$$

where *w*_*j*_ is the PF synaptic weight at compartment *j*, and *A*_1_, *A*_2_, *A*_3_ are constants set at 0.9, 0, and 0.001, respectively. Here, we set *A*_2_ = 0 to simulate LTD. If *w*_*j*_ became negative by learning, it was set to zero. On the contrary, if it became more than 12.87 nA, it was fixed to 12.87 nA. The LTD between PF–PC is caused by α-amino-3-hydroxy-5-methyl-4-isoxazolepropionic acid (AMPA) receptors on the surface of the membrane that are introduced again to the inner surface of the membrane over time. Physiologically, the *A*_3_ can be regarded as a representation of the function of AMPA receptors that have gone under the membrane and appear on the surface once more (Hansel et al., 2001).

The exact procedure was as follows. First, we identified sequences that evoked responses in either IN or OUT direction while turning learning off. Next, we turned on the learning, and applied each sequence again, which was followed by CF stimulus immediately with a 2-ms delay. The entire simulation period was set at 5,000 ms. Then, we applied each sequence once again while turning the learning off, and examined whether the stimulation evoked somatic spikes. Finally, we searched sequences that changed the direction to evoke responses before and after learning.

### 2.5. Numerical simulation

We used an implementation of a previous PC model (Kobayashi et al., 2021). It applied an explicit method and enabled us to calculate faster than conventional implicit methods; the implicit methods took about 3,500 s for a 5,000-ms trial, and the explicit method on a GPU could finish in about 250 s.

Our study used an NVIDIA DGX station (NVIDIA, 2017) composed of Intel Xeon E5-2698 v4 2.2 GHz and 4 GPU Tesla V100 32 GB. The OS was Ubuntu 18.04, and the CUDA version was 10.0. The computational environment was the same for all of the simulations.

## 3. Results

### 3.1. Discrimination of sequences

**Figure 4:**
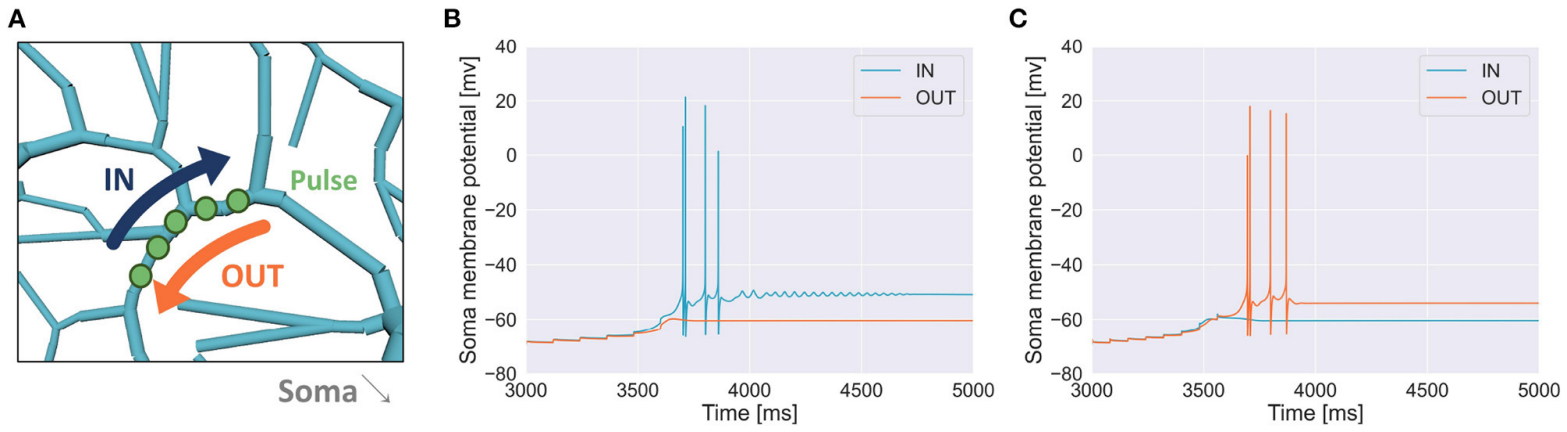
**(A)** Schema of stimulation. A segment of spiny dendrites was stimulated sequentially in either IN direction (blue) or OUT direction (orange), where the number of compartments was 5, time interval for each stimulation was 120 ms and synaptic weight (or pulse amplitude) was 7.920 nA. The stimulated site was shown in Figure 1. **(B)** Membrane potential in the soma recorded during the stimulation in IN direction (blue) and that in OUT direction (orange) as in **(A)**, where the number of pulses was 6. **(C)** Another example when a different segment was stimulated, where the number of compartments was 8, interval was 80 ms and synaptic weight was 6.930 nA.

To examine whether a PC could discriminate a temporal input sequence that stimulates dendritic compartments aligned in one dimension one by one sequentially with another sequence that stimulates the same compartments but in the opposite direction, first, we chose a short segment of spiny dendrites (Figure 4A) and fed a pulse to each dendritic compartment in the segment sequentially in either IN direction or OUT direction with a certain temporal interval. We found that a sequence in IN direction was able to evoke somatic spikes while that in OUT direction was not (Figure 4B). We also found that for a different segment, a sequence in OUT direction evoked somatic spikes but that in IN direction failed (Figure 4C). These results suggest that depending on the location of dendritic segments, the PC model showed selectivity on the direction of sequential stimulation.

**Figure 5:**
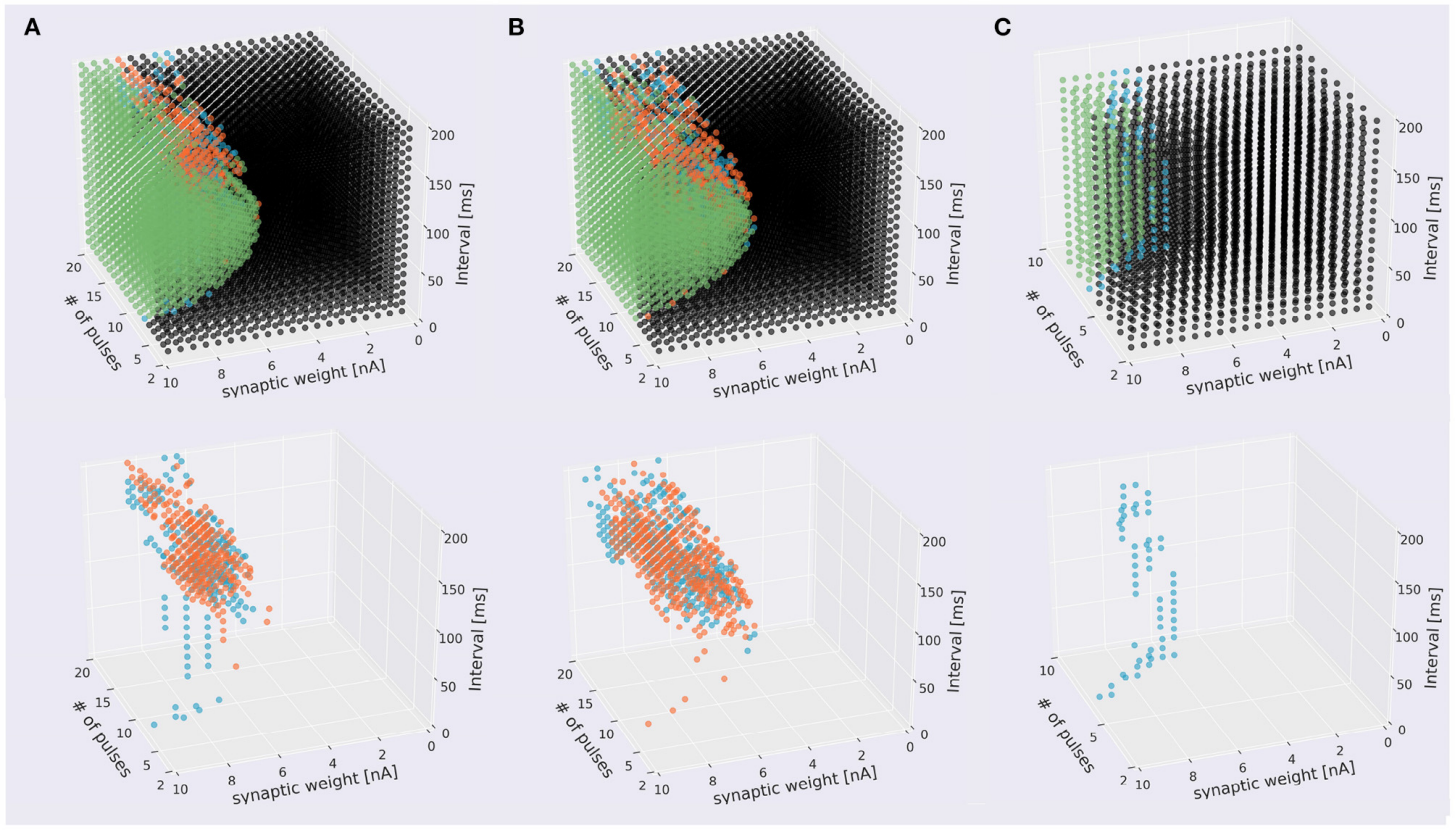
Scatter plots of stimuli that evoked different responses. The top panels four types of stimuli that evoked somatic response when stimulated in IN direction (blue), OUT direction (orange), both directions (green), and that did not evoke responses (black). The bottom figures omitted the plots of non-discriminated sequences. **(A)** Sequences starting from spiny dendrite. A 2,328 sequences induced spikes in both stimulus directions, 199 sequences were IN-only, 322 sequences were OUT-only, and 4,751 did not induce spikes in both directions. **(B)** Sequences starting from smooth dendrite. A 1,779 sequences caused spikes in both directions, 304 sequences were IN-only, 381 sequences were OUT-only, and 5,136 did not induce spikes in both directions. **(C)** Sequences starting from main dendrite. A 286 sequences induced spikes in both directions, 59 sequences were IN-only, and no sequences were OUT-only, A 2,455 sequences did not cause spikes in both directions.

Next, we tried to inject at three locations for different types of dendrites. In the pulse sequences starting from a compartment of spiny dendrites, the PC model discriminated stimulus direction. We injected 7,600 sequences having different strengths or synaptic weights, intervals and times (19 times × 20 interval × 20 strength) by two directions and presented these distribution in the form of a cube (Figure 5A, Supplementary Video 2A). The soma responses were classified by 4 types: emitting spikes both directions, only IN direction, only OUT direction, and no spikes both directions. These results show that the PC model discriminated the directions of the stimuli and represented the responses in the form of spiking. Similarly, in the pulse sequences starting from a compartment of smooth dendrites, the PC model also could discriminate stimulus directions (Figure 5B, Supplementary Video 2B). Finally, in main dendrites, we injected 2,800 (7 times × 20 interval × 20 strength) sequences having different strengths, intervals and times. As a result, we observed spike firing in only the IN direction (Figure 5C, Supplementary Video 2C). We summarized these results in Figure 5, which shows that all dendrite types had direction sensitivity. With respect to types of dendrites, there was no significant difference between spiny dendrites and smooth dendrites, but the main dendrites had only sequences inducing spikes in the IN direction: no sequences caused spikes in the OUT direction. This result suggests that main dendrites have relatively low direction sensitivity. The main dendrites are thick and so the surface is wide, suggesting that the intracellular currents on the main dendrites can be leaked more than the spiny and smooth dendrites. To overcome the passive leak and let the soma emit spikes, one should feed external currents from the distal part to the soma (IN direction), rather than the opposite direction (OUT direction). On the other hand, the spiny dendrites—elaborately spreading like trees and receiving inputs from PF—has high direction sensitivity so dendritic nonlinear forms seem to support better direction sensitivity.

**Figure 6:**
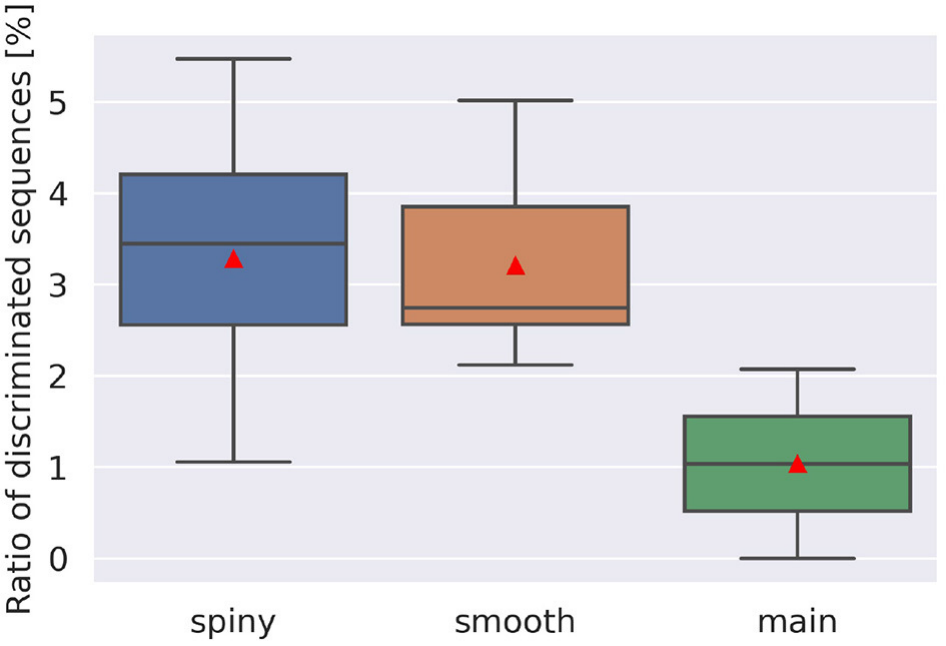
Ratio of discriminated sequences. The discriminated sequences consisted of the pulse sequences spiking in IN and OUT. Red triangles are averaged values. Note that we had only one cube for main dendrites, where stimulation started from compartment number 1,573, whereas for spiny and smooth dendrites, the cube numbers were 13 and 7, respectively.

To examine the discrimination ability of each type of dendrite, we conducted additional simulations while changing the start point of stimulation, and obtained 21 cubes as in Figure 5A, where 13 cubes started from various spiny dendrites, seven cubes from smooth dendrites, and one cube from the main dendrites. First, we defined two types of pulse sequences from soma responses; discriminated sequences contained the sequences causing spikes in IN and OUT directions, and non-discriminated sequences consisted of the sequences causing spikes (or none) in both directions. Next, the numbers of response types were counted (Figure 6). Because all types had discriminated sequences, these results suggest that the PC models were capable of discriminating spatiotemporal sequences regardless of the types of compartments. In terms of dendritic types, spiny and smooth dendrites spreading like complex trees showed better direction sensitivity than main dendrites, which provides additional evidence for revealing the effects of dendritic nonlinearity on better computational ability. Besides, the PC delayed rectifier (Kdr) was included in only main dendrites and soma, so this channel may play the role of reducing the effect of dendritic nonlinearity and finally filtering the outputs of the PC.

A more careful examination show that direction selectivity was observed when input stimuli are composed of 4–20 pulses with intervals of 10–200 ms (spiny), 5–20 pulses with intervals of 20–200 ms (smooth), and 6–8 pulses with intervals of 10–200 ms (main). From these observations, the shortest stimulus spans 60 ms, whereas the longest one 4,000 ms. Furthermore, for each dendritic location, the mean direction selective stimulus is 14.06 pulses that have the synaptic weight of 6.542 nA with interval of 129.1 ms (spiny), 14.96 pulses that have the synaptic weight of 6.472 nA with interval of 126.0 ms (smooth), and 7.431 pulses that have the synaptic weight of 7.374 nA with interval of 98.79 ms (main), respectively.

### 3.2. Role of Ca^2+^ channels

**Figure 7:**
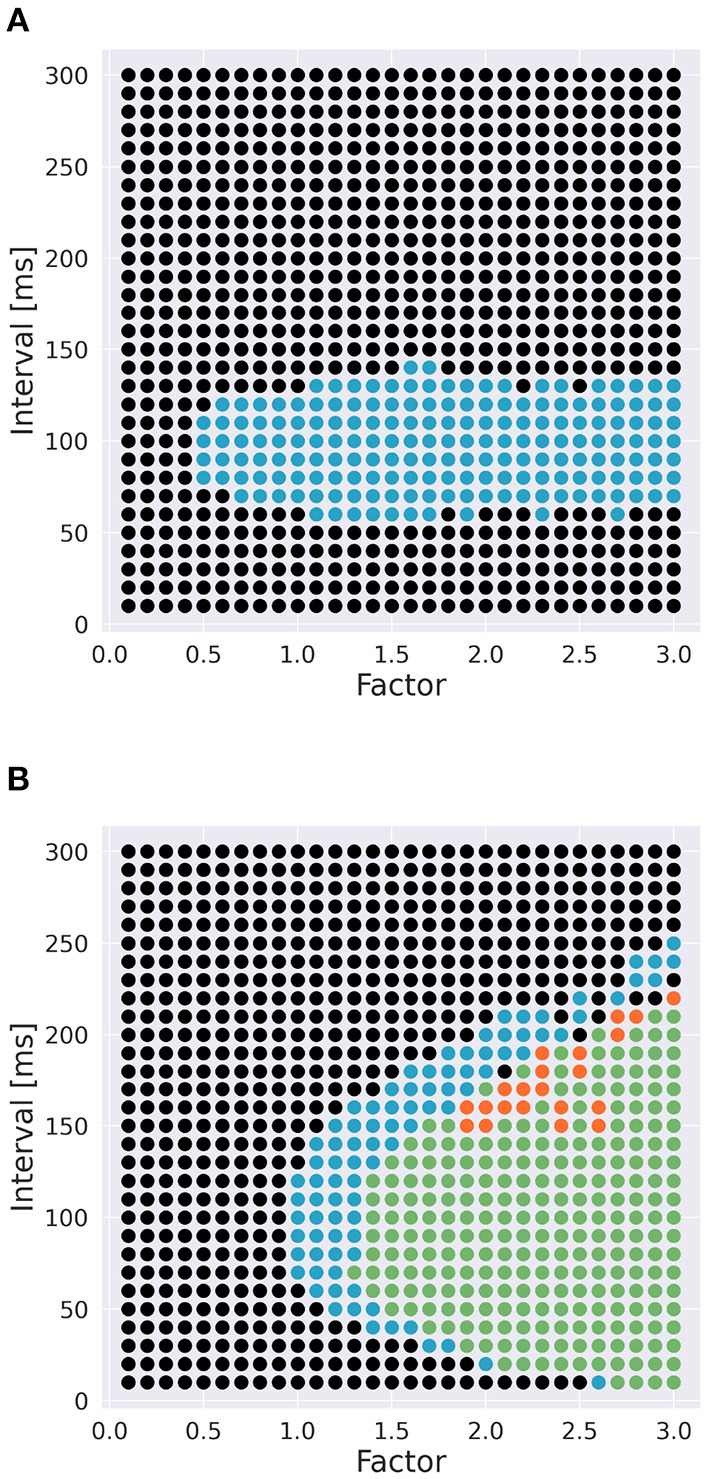
Effect of time constants of Ca^2+^ channels [**(A)**: CaP channel; **(B)**: CaT channel] and interval on sequence discrimination. Time constants were multiplied by a factor *f* (Figure 3). Colors are the same as in Figure 5. The number of pulses was 6, and synaptic weight was 6.633 nA.

To investigate the role of Ca^2+^ channels, we changed inactivation time constants of CaP and CaT (Figure 3). When we changed the time constant of CaP, the range of intervals that are effective to cause spikes remained constant unless *f* < 0.5 (Figure 7A). Moreover, only stimuli in IN direction evoked spikes. These results suggest that varying the time constant of CaP would not affect the discrimination ability. On the other hand, in the case of CaT, in general, larger (or smaller) *f* (i.e., slower or faster dynamics) evoked spikes in both directions or no spikes, respectively. At the border of the spiking/non-spiking, however, there is a small region that evoked spikes in either IN or OUT direction. Furthermore, the region was curved, suggesting that the relationship between the value of *f* and the interval that evoked spikes was nonlinear. We would imply that the range of time constants that caused spiking (40–60 ms) is in the same order of that of stimulus interval that caused spiking (50–150 ms). In fact, the upper half of the domain in which PC emit spikes in IN or OUT direction elongates linearly. These observation suggest that the time constant of CaT plays an essential role for sequence discrimination.

### 3.3. Learning of sequence direction

**Figure 8:**
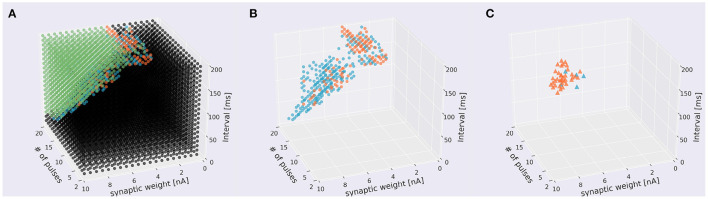
Distribution of sequences under the presence of learning. **(A)** Scatter plot. A 1,310 sequences emit spikes in both directions, 235 were IN-only, 106 were OUT-only, and 5,949 sequences did not cause in either direction. Conventions as in the top panel of Figure 5A. **(B)** The same plot leaving only direction-sensitive sequences (blue: OUT direction; orange: IN direction) as in the bottom panel of Figure 5A. **(C)** Sequences that were able to be changed the direction before and after learning. Blue and orange dots represent sequences that changed the direction from IN to OUT and from OUT to IN to emit spikes, respectively.

To invesitgate whether the direction of sequences can be reversed, we incorporated a simulated LTD mechanism, which was modeled as an STDP rule depending on the timing of each pulse stimulus (i.e., PF stimulus) and that of a simulated CF stimulus, to update PF–PC synaptic weights [Equation (10)]. Specifically, for each presentation of a sequence, CF stimulus was fed immediately after the presentation. First, we studied how the distribution of sequences that evoked responses was changed by learning (Figure 8A, Supplementary Video 3A). We found that sequences in IN direction became sensitive to the choice of number of pulses at around 10 (Figure 8B, Supplementary Video 3B) compared with those without learning (Figure 5B), and had distributed synaptic weights. On the other hand, the distribution of sequences in OUT direction became fewer. These results suggest that learning enhances sequence discrimination only in IN direction. Finally, by applying reversed version of sequences that evoked responses, we examined whether a PC model can learn to reverse the sequence direction. As a result, a limited number of sequences were able to reverse the direction from IN to OUT and OUT to IN (Figure 8C, Supplementary Video 3C). By examining the stimulus parameters, we found that such reversible stimuli were distributed locally, suggesting that stimuli must be set appropriately to control the direction.

## 4. Discussion

### 4.1. Sequence discrimination in cerebellar PCs

We examined whether a detailed morphological model of cerebellar PCs could respond differently to two sequences of dendritic stimulation by injecting short pulses on dendrites at various locations and timings. We recruited the same dendritic compartments as the target of the stimulation, and injected short pulses in one of two directions: from distal to proximal dendrites or the opposite. Owing to the complex morphological structure of dendrites and various ion channels distributed across the dendrites, our model responded differently to the direction of stimulation, suggesting that the model exhibited direction sensitivity on the temporal order of the stimulus (Figures 4, 5). In other words, our PC model was capable of sequence discrimination for input stimuli. Furthermore, the parameter range for discriminable sequences fall within the range of hundreds of milliseconds up to a few seconds, which seems to be appropriate for cerebellum-dependent motor learning (Ivry and Spencer, 2004).

The ability of direction sensitivity or sequence discrimination is found even in linear passive cables (Rall, 1964), if the temporal interval of each pulse injection is as short as a few milliseconds so that the cables can bridge the successive pulse injections temporally and integrate them. In contrast, we set the temporal interval much longer up to 100 ms, which cannot be bridged by passive cables. For such stimuli with long time intervals, nonlinear integration over active cables is necessary. Branco et al. (2010) demonstrated that cortical layer 2/3 pyramidal neurons showed direction sensitivity for stimuli with longer intervals, where the entire duration was set at about 100 ms. They concluded that NMDARs and intracellular Ca^2+^ play an important role in direction sensitivity. This is natural, because the time constants of NMDAR-mediated excitatory postsynaptic potentials (EPSPs) and Ca^2+^ dynamics are on the order of one hundred milliseconds. However, it has been shown that PCs in adult animals do not show NMDAR-mediated currents for PF inputs (Perkel et al., 1990; Llano et al., 1991), but have Ca^2+^-mediated currents (CaP and CaT) with long time constants as long as a few hundred milliseconds. We confirmed that the direction sensitivity was spoiled and so the sequence discrimination was disrupted when the Ca^2+^-mediated currents were blocked (Figure 7). These results suggest that intracellular Ca^2+^ dynamics is an important factor for individual neurons to perform complex computation, including synaptic plasticity (e.g., Zucker, 1999).

On the other hand, the present study does not deny potential roles of NMDARs on PCs in information processing. For example, Piochon et al. (2010) reported that NMDARs are expressed on CF synapses on PCs and involved in controlling synaptic gain. Galliano et al. (2018) over expressed NMDARs on PF–PC synapses by genetic manipulation, and observed that larger NMDAR-mediated EPSPs that blocked long-term potentiation (LTP) at PF–PC synapses, suggesting that lack of NMDARs allows LTP to contribute to motor learning. Schonewille et al. (2021) reported involvement of presynaptic NMDARs activation at PF terminals to the synaptic plasticity. These results support the involvement of NMDAR-related contribution in the computation of PCs.

### 4.2. Sequence learning in cerebellar PCs

The present study also demonstrated that when the sequential dendritic stimulation was paired with a simulated CF stimulus, our PC model changed the preferred direction of the temporal order of the dendritic stimulation (Figures 4, 5), although the parameter space in which direction selectivity can be reversed is rather small. One reason might be that we tried only one learning trial for each stimulus. If we repeat many learning trials, we would be able to obtain a wider parameter space. Meanwhile, this dynamical change was realized by changing the effective amplitude of each dendritic pulse injection, which would correspond to changing the synaptic weight of a PF input, induced by the timing of each pulse injection and that of the large depolarization, representing CF stimulation. In other words, this paired stimulation attempted to simulate LTD and LTP at PF–PC synapses induced by the occurrence or nonoccurrence of CF stimulation in a temporal manner (i.e., spike timing-dependent plasticity).

Temporal dependency of paired stimulation of PFs and CF for LTD induction has been studied intensively. After establishing LTD induction techniques in slice experiments (Ito, 1989), researchers investigated the dependency of LTD induction on the temporal interval between the PF and CF stimulation. Early studies demonstrated that LTD induction was maximal when PF stimulation was advanced for 250–300 ms to CF stimulation, and the induction level was dependent on the PF–CF interval (Karachot et al., 1994; Chen and Thompson, 1995). More recently, Suvrathan et al. (2016) assessed the temporal dependence across various cerebellar cortical areas comprehensively, and reported that the best temporal interval was different across cortical areas. These results suggest that PF–PC LTD is induced in an STDP manner, which is consistent with the assumption made in the present study.

On the other hand, theoretical and computational studies have been repeatedly demonstrating the importance of STDP rules for temporal information processing including sequence learning (e.g., Wörgötter and Porr, 2005), where intracellular Ca^2+^ plays essential roles in realizing STDP. The present computational study also supports the importance of STDP rules in the context of the cerebellar computation.

### 4.3. Potential roles of dendritic computation in PCs on cerebellar functions

Our findings on sequence learning suggest that PCs do not just exhibit direction sensitivity on the temporal order of PF stimulation, but also are able to learn the preferred direction of the temporal order.

Traditionally, cellular and circuit mechanisms of temporal information processing in the cerebellum have been studied experimentally in Pavlovian delay eyeblink conditioning, where temporal codes are assumed to be represented by granule cells and the temporal information is read out by PCs (McCormick and Thompson, 1984; Mauk and Donegan, 1997). Moreover, a number of theoretical studies have proposed different mechanisms to generate such temporal codes (Yamazaki and Tanaka, 2009). Those studies, however, have assumed that individual neurons are simple elements that cannot perform complex functions by dendritic computation. In contrast, a few studies have proposed other types of timing mechanisms on single PCs rather than network mechanisms (Fiala et al., 1996; Majoral et al., 2020). The present study seems consistent with a view that single PCs might be sufficient for temporal information processing in the cerebellum (Johansson et al., 2014).

Another direction of dendritic computation by a single PC is pattern recognition based on simple spike pause controlled by PF–PC LTD. While capacity of information storage and pattern recognition by PCs' dendrites have been studied theoretically (Brunel et al., 2004; Steuber et al., 2007; De Schutter and Steuber, 2009; Clopath et al., 2012; Sezener et al., 2022) demonstrated that the duration of simple spike pause represents information through nonlinear temporal integration of PF stimuli, suggesting a form of temporal information processing using PC dendrites. Our study is related to these studies in the sense that we incorporated temporal sequence in PF stimuli and examined the difference of responses of an individual PC. By considering the temporal domain, capacity of information storage of PCs might be enhanced compared with the classical perceptron as a model of the cerebellum (Marr, 1969; Albus, 1971; Ito, 1984).

### 4.4. Dendritic computation by other detailed morphological models

Besides the cerebellar PC model that we investigated for sequence discrimination, detailed morphological models have demonstrated various functions while harnessing dendritic computation. Branco et al. (2010) and Bicknell and Häusser (2021) originally demonstrated the capability of the sequence discrimination by cortical Pyramidal cells experimentally and computationally. Gidon et al. (2020) reported that cortical Pyramidal cells in humans could perform a boolean logic function called XOR, which is a versatile boolean operations, experimentally and computationally. Moldwin and Segev (2020) have proposed that cortical Pyramidal cells could act as a multi-layer perception, a versatile supervised learning machine. In the context of deep learning, Lillicrap et al. (2020) have proposed how to implement back propagation, which is the learning mechanism essential for deep neural networks, based on dendritic computation, and Payeur et al. (2021) successfully implemented back propagation. These results strongly support the view that dendritic computation is a powerful means for machine learning.

### 4.5. Numerical simulation of detailed morphological models

To examine sequence discrimination capacity, we had to repeat the same simulation many times while varying the stimulus parameters such as the temporal order, stimulation interval, and the number of pulses (Figure 5). To conduct such a large number of simulations, we used our own implementation of the PC model rather than conventional and publicly available ones implemented on simulation software (De Schutter and Bower, 1994; Roth and Häusser, 2001). Although implementing detailed morphological models is tedious yet time consuming, we had two reasons to do this.

The first reason is on the efficiency of numerical methods on modern computers. Dendritic computation must be assessed by using detailed morphological computational models of neurons. Because numerical simulation for such models is complicated and time consuming, one uses dedicated simulation software such as GENESIS (Wilson et al., 1989), NEURON (Hines and Carnevale, 1997), and Arbor (Akar et al., 2019). These software programs use implicit methods such as a backward Euler method and Crank-Nicolson method for solving partial differential equations (PDEs) that describe the current flow across dendrites. Implicit methods are unconditionally stable methods so that the calculation is always consistent. However, to achieve the stability, those methods have to solve large simultaneous equations whose size is in proportional to the number of the dendritic compartments for each cell for each simulation step. Here, a problem for modern computers is that solving simultaneous equations results in a large amount of memory access, which could be the bottleneck of efficient numerical simulation (Kobayashi et al., 2021). Instead, our implementation uses an explicit method developed specifically for diffusion equations including cable equations, which reduces the amount of memory access substantially (Kobayashi et al., 2021). Eventually, we were able to speed up the computation by about 15 times compared with an implicit method.

The second reason is about the performance trend of supercomputers. At present, the state-of-the-art large-scale simulations build networks comprising tens of thousands of detailed morphological neuron models (Markram et al., 2015; Billeh et al., 2020). Such large-scale simulation, however, takes a long time even with supercomputers, because on modern supercomputers, the memory access becomes the bottleneck more than the computational power (Machanick, 2002). To address this problem, we propose the use of explicit methods for simulation of detailed morphological neurons and networks as in the present study.

### 4.6. Limitations

Several limitations exist in the present study. First, we implemented and used a rather old PC model (De Schutter and Bower, 1994). The model has been revised significantly to date, and the current version contains more ion channels and realistic diffusion dynamics (Zang et al., 2020; Zang and De Schutter, 2021). Our study suggests that slow Ca^2+^ dynamics plays an essential role in sequence discrimination, and this conclusion would not change even in the more realistic models. Nevertheless, it would be of interest to examine these more realistic models. In particular, our model does not emit simple spikes spontaneously, which is suitable to see the responses related to the discrimination, whereas the current version does. To discriminate sequences under the presence of spontaneous activity, we must consider the response of PCs based on the pause rather than the firing (De Schutter and Steuber, 2009; Zang et al., 2020; Zang and De Schutter, 2021). Thus, the second limitation is that pause-based stimulus discrimination should be assessed.

## 5. Conclusion

Owing to their remarkable dendrites, individual cerebellar PCs could be capable of complex spatiotemporal information processing. Considering such dendritic computation will shed new light on cerebellar information processing and cerebellar learning.

## Data availability statement

Publicly available datasets were analyzed in this study. This data can be found here: https://github.com/kaaya542/Dicrimination-and-learning-of-temporal-input-sequences-in-a-cerebellar-Purkinje-cell-model.

## Author contributions

YY and TY conceived and designed the research. YY conducted preliminary computer simulation. KT performed all simulations and analyzed data. TK contributed the simulation code of the PC model. KT, TK, RK, and TY determined analysis methods and discussed the draft and revised for the final version. KT and TY wrote the original draft. All authors contributed to the article and approved the submitted version.
