# Supplementary material for: Discrimination and learning of temporal input sequences in a cerebellar Purkinje cell model
Source: Front Cell Neurosci. 2023 Feb 2;17:1075005. doi: 10.3389/fncel.2023.1075005 (PMC9932327; doi:10.3389/fncel.2023.1075005)
Supplement: Supplementary file 4 [file Data_Sheet_1.PDF]

Supplementary Material

1 SUPPLEMENTARY TABLES AND FIGURES

Table S1. Details of kinetic equations for ion channels

channel name	gate variable	multiplier	α [1/ms]	β [1/ms]
NaF	m	3	$35.0/\exp(-(v+5.0)/10.0)$	$7.0/\exp((v+65.0)/20.0)$
	h	1	$0.226/(1+\exp((v+80.0)/10.0))$	$7.5/\exp(-(v-3.0)/18.0))$
NaP	m	3	$200.0/(1+\exp(-(v-18.0)/16.0))$	$25.0/(1+\exp((v+58.0)/8.0))$
CaP	m	1	$8.5/(1+\exp(-(v-8.0)/12.5))$	$35.0/(1+\exp((v+74.0)/14.5))$
	h	1	$0.0015/(1+\exp((v+29.0)/8.0))$	$0.0055/(1+\exp(-(v+23.0)/8.0))$
CaT	m	1	$2.60/(1+\exp(-(v+21.0)/8.0))$	$0.18/(1+\exp((v+40.0)/4.0))$
	h	1	$0.0025/(1+\exp((v+40.0)/8.0))$	$0.19/(1+\exp(-(v+50.0)/10.0))$
KA	m	4	$1.40/(1+\exp(-(v+27.0)/12.0))$	$0.49/(1+\exp((v+30.0)/4.0))$
	h	1	$0.0175/(1+\exp((v+50.0)/8.0))$	$1.30/(1+\exp(-(v+13.0)/10.0))$

Table S2. Details of kinetic equations for ion channels

channel name	gate variable	multiplier	$\tau_x(v)$ [ms]	$x_0(v)$
Kh1	m	1	7.6	$0.8/(1+\exp((v+82.0)/7.0))$
Kh2	m	1	36.8	$0.2/(1+\exp((v+82.0)/7.0))$
Kdr	m	1	$\begin{cases} 1200.0 & (v < -25.0) \\ 10.0 & (\text{ otherwise }) \end{cases}$	$\frac{\alpha_{\mathrm{Kdr}}}{\alpha_{\mathrm{Kdr}}+5.0\exp(-(v+127.0)/30.0)}$
			$\alpha_{\mathrm{Kdr}} = \begin{cases} \frac{(0.0235\times(v-8.0))}{(\exp(-(v-8.0)/12.0)-1.0)} & (\lvert v-8.0\rvert > 10^{-6}) \\ 0.282 & (\text{otherwise}) \end{cases}$	
KM	m	1	$\frac{60.61}{\exp((v+35.0)/20.0)+\exp(-(v+35.0)/20.0))}$	$1.0/(1+\exp((v+25.0)/4.0))$
KC	m	1	$\frac{1}{7.5+0.110/\exp((v-35.0)/14.9)}$	$\frac{7.5}{7.5+0.110/\exp((v-35.0)/14.9)}$
	h	2	10.0	$1.0/(1.0+4.00/[\mathrm{Ca}])$
K2	m	1	$\frac{1}{25.0+0.075/\exp((v+25.0)/6.0)}$	$\frac{25.0}{25.0+0.075/\exp((v+25.0)/6.0)}$
	h	2	10.0	$1.0/(1.0+0.20/[\mathrm{Ca}])$
